# Viral Escape from HIV-1 Neutralizing Antibodies Drives Increased Plasma Neutralization Breadth through Sequential Recognition of Multiple Epitopes and Immunotypes

**DOI:** 10.1371/journal.ppat.1003738

**Published:** 2013-10-31

**Authors:** Constantinos Kurt Wibmer, Jinal N. Bhiman, Elin S. Gray, Nancy Tumba, Salim S. Abdool Karim, Carolyn Williamson, Lynn Morris, Penny L. Moore

**Affiliations:** 1 Centre for HIV and STIs, National Institute for Communicable Diseases (NICD), of the National Health Laboratory Service (NHLS), Johannesburg, South Africa; 2 Faculty of Health Sciences, University of the Witwatersrand, Johannesburg, South Africa; 3 Centre for the AIDS Programme of Research in South Africa (CAPRISA), University of KwaZulu-Natal, Durban, South Africa; 4 Institute of Infectious Disease and Molecular Medicine (IIDMM) and Division of Medical Virology, University of Cape Town and NHLS, Cape Town, South Africa; University of Zurich, Switzerland

## Abstract

Identifying the targets of broadly neutralizing antibodies to HIV-1 and understanding how these antibodies develop remain important goals in the quest to rationally develop an HIV-1 vaccine. We previously identified a participant in the CAPRISA Acute Infection Cohort (CAP257) whose plasma neutralized 84% of heterologous viruses. In this study we showed that breadth in CAP257 was largely due to the sequential, transient appearance of three distinct broadly neutralizing antibody specificities spanning the first 4.5 years of infection. The first specificity targeted an epitope in the V2 region of gp120 that was also recognized by strain-specific antibodies 7 weeks earlier. Specificity for the autologous virus was determined largely by a rare N167 antigenic variant of V2, with viral escape to the more common D167 immunotype coinciding with the development of the first wave of broadly neutralizing antibodies. Escape from these broadly neutralizing V2 antibodies through deletion of the glycan at N160 was associated with exposure of an epitope in the CD4 binding site that became the target for a second wave of broadly neutralizing antibodies. Neutralization by these CD4 binding site antibodies was almost entirely dependent on the glycan at position N276. Early viral escape mutations in the CD4 binding site drove an increase in wave two neutralization breadth, as this second wave of heterologous neutralization matured to recognize multiple immunotypes within this site. The third wave targeted a quaternary epitope that did not overlap any of the four known sites of vulnerability on the HIV-1 envelope and remains undefined. Altogether this study showed that the human immune system is capable of generating multiple broadly neutralizing antibodies in response to a constantly evolving viral population that exposes new targets as a consequence of escape from earlier neutralizing antibodies.

## Author Summary

Four sites of vulnerability for broadly neutralizing antibodies to HIV-1 have been identified thus far. How these broadly reactive antibodies arise, and the host-pathogen interactions that drive the affinity maturation necessary for neutralization breadth are poorly understood. This study details the sequential development of three distinct broadly neutralizing antibody responses within a single HIV-1 infected individual over 4.5 years of infection. We show how escape from the first wave of antibodies targeting V2 exposed a second site that was the stimulus for a new wave of glycan dependent broadly neutralizing antibodies against the CD4 binding site. These data highlight how antibody evolution in response to viral escape mutations served to broaden the host immune response to these two epitopes. Finally, we document a third wave of neutralization that targets an undefined epitope that did not appear to overlap with the four known sites of vulnerability on the HIV-1 envelope. These data support the design of templates for sequential immunization strategies aimed at increasing neutralization breadth through the recognition of multiple epitopes and their immunotypes.

## Introduction

Neutralizing antibodies are the principal correlate of protection for most preventative vaccines. Designing suitable vaccine immunogens to elicit these types of antibodies has been relatively simple for conserved pathogens such as smallpox and other DNA viruses. For more diverse pathogens like HIV-1, the neutralizing antibodies elicited by vaccination or during natural infection are largely strain-specific and therefore would not be protective against globally circulating viral variants [1]–[5]. The HIV-1 envelope glycoprotein spikes mediate viral entry and are the sole targets for neutralizing antibodies. The spikes are trimeric, made up of three non-covalently associated gp41-gp120 heterodimers, each with a conserved core that mediates infection of CD4^+^ T-cells. Functionally conserved sites are protected by extensive glycosylation, and large solvent exposed hypervariable structures (the V1–V5 loops, and the α2-helix in C3) [6]. All HIV-1 infected individuals develop strain-specific neutralizing antibodies which target these sequence variable regions, but only a quarter develop broadly neutralizing antibodies [7]–[11], which will likely be needed for a preventative HIV-1 vaccine. To engineer an envelope immunogen that can specifically elicit these antibodies, the HIV-1 vaccine research field has adopted a strategy based largely on rational design: identifying the targets for these broadly cross-reactive antibodies, and elucidating the pathways that promoted their development.

Plasma mapping strategies and the isolation of monoclonal antibodies have defined four major targets for broadly neutralizing antibodies on the HIV-1 glycoprotein [7]–[10], [12]–[20]. The CD4 binding site (CD4bs) of gp120 and the membrane proximal external region (MPER) of gp41 are glycan independent epitopes, while the V1/V2 sub-domain and the co-receptor/V3 site on gp120 are sites of vulnerability for glycan binding antibodies (predominantly at positions N156/N160 and N301/N332 respectively) [14]–[16]. Both CD4bs antibodies and co-receptor/V3 antibodies bind well to monomeric gp120, while MPER antibodies bind to a linear peptide in gp41. This makes it possible to adsorb out their neutralization activity from plasma with various recombinant proteins. In contrast the epitope for V2 antibodies (such as PG9/16) consists of two anti-parallel β-sheets (B- and C- strands) of a Greek key motif, and the glycans therein, that is preferentially formed on the native trimer. This region is critically important for the gp120-gp120 interactions that stabilize the envelope glycoprotein spike in its unliganded conformation, and therefore cannot be readily adsorbed [16], [21]. Various sub-epitopes within each of these four major sites of vulnerability have also been identified through subtle differences in the mechanism of neutralization [22]. For instance antibodies targeting the CD4bs can be sub-divided into two groups: those that are sensitive to the D368A and/or E370A mutations in the CD4 binding loop α3 (such as VRC01); and those that are dependent on amino acids D474, M475, and/or R476 in α5 termed CD4bs/DMR (such as HJ16) [23]–[26]. Despite this detailed knowledge, epitope mapping strategies have failed to identify the neutralization targets in a subset of plasma samples [7]–[9], [17], [19], [27], [28]. The antibodies mediating breadth in these samples could target sub-epitopes within one of the four sites of vulnerability or they may target entirely novel epitopes.

In the CAPRISA 002 Acute Infection Cohort, we previously identified seven individuals with broadly neutralizing antibodies. In five cases we were able to map the plasma antibody specificities to known epitopes (two targeted N332, two the V2 epitope, and one the MPER) [7]. In this study we have focused on one of the individuals (CAP257) for which the target was undefined. Heterologous and autologous neutralization data as well as viral sequences from longitudinal samples were used to identify the epitopes for CAP257 broadly neutralizing antibodies. We showed that heterologous neutralization in CAP257 was conferred by three distinct, sequentially occurring antibody waves, two of which were mapped to epitopes in V2 and the CD4bs respectively. While individuals with more than one broadly neutralizing antibody specificity have been previously identified [29]–[31], there is little information on how the dynamic relationship between host and pathogen contributed to the development of antibodies targeting multiple epitopes. We have shown previously that escape from strain-specific neutralizing antibodies can drive the formation of epitopes for broadly neutralizing antibodies [32]. Here we found that viral escape from broadly neutralizing antibodies targeting V2 promoted the development of a second broadly neutralizing antibody response targeting a glycan dependent epitope in the CD4bs. We also identified early escape mutations from both the V2 and CD4bs antibodies that drove an increase in the neutralization breadth of CAP257 plasma. These findings have implications for the design of HIV-1 vaccine antigens and sequential immunization strategies.

## Results

### The broadly neutralizing activity of CAP257 develops in three distinct waves

**Figure 1:**
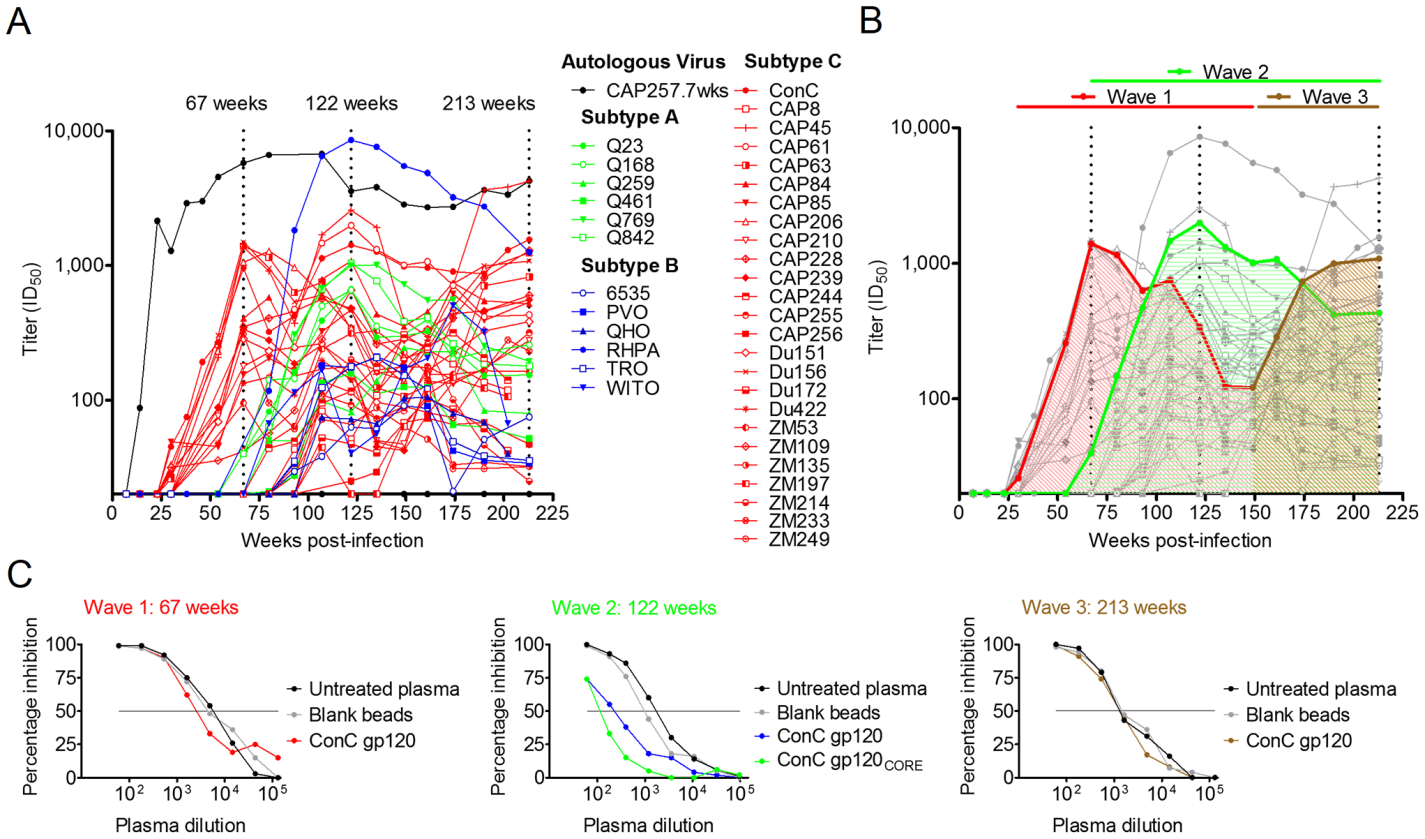
CAP257 broadly neutralizing antibodies develop sequentially in three distinct waves. **A**) Longitudinal neutralization of the autologous CAP257 virus (black) and 37 heterologous viruses neutralized by CAP257 plasma at titers >1∶100. The ID_50_ titers (y-axis) are shown versus weeks p.i. (x-axis). Three peaks in heterologous neutralization titers at 67, 122, and 213 weeks p.i. are indicated with dotted lines. Heterologous viruses are colored according to subtype (A = green, B = blue, C = red). **B**) A summary of the three waves of heterologous neutralization defined by a representative virus, superimposed over the neutralization kinetics shown in Figure 1A. Wave 1 was subtype C specific and is colored red. Wave 2 neutralized viruses from all three clades and is colored green. Wave 3 is colored brown. **C**) Adsorption of heterologous neutralization at the peak of each of the three waves. Percentage inhibition (y-axis) is shown versus plasma dilution (x-axis). Untreated plasma is shown in black, blank beads in grey and beads coated with recombinant proteins are shown in red (wave 1), blue/green (wave 2) or brown (wave 3).

We have previously described the development of neutralization breadth in CAP257 using longitudinal plasma samples from HIV-1 seroconversion to three years post-infection (p.i.) [7]. Here, we extended this analysis until the start of anti-retroviral therapy at four and a half years p.i. (Figure 1A). Longitudinal plasma was tested against the autologous CAP257 virus amplified from the earliest available time point (7 weeks p.i.), the subtype C consensus sequence (ConC) [33], 4 Tier 1b viruses, and 39 Tier 2 viruses [34]. Autologous neutralizing antibodies appeared by 14 weeks of infection with a peak titer at two years of 1∶6,754. This was followed by the neutralization of heterologous viruses 30 weeks after infection. CAP257 neutralized 84% of the heterologous viruses at three years (174 weeks) with neutralization breadth of 100% against subtype A (6/6 viruses), 96% against subtype C (25/26 viruses, including ConC), and 50% against subtype B (6/12 viruses). The titers of these broadly neutralizing antibodies peaked and waned in three separate waves.

The first wave of neutralization breadth (typified by CAP63) peaked at 67 weeks p.i. with a maximum titer of 1∶1,493 and exclusively neutralized subtype C viruses (Figures 1A and 1B – red curves). Wave 1 titers dropped to as low as 1∶145 by 149 weeks of infection. As this early heterologous neutralization began to wane, CAP257 plasma gained the capacity to neutralize additional subtype C viruses as well as several subtype A and B viruses (Figures 1A and 1B – blue and green curves). This second wave (typified by Q842) peaked at 122 weeks p.i. with titers as high as 1∶8,565 against RHPA that dropped to 1∶1,254 by 213 weeks of infection. Finally, a third wave of heterologous neutralization (represented by Du156) appeared by 149 weeks p.i. and peaked at 213 weeks p.i. (Figure 1B – brown curve). This third wave was also largely subtype C specific. These data suggested that the neutralization breadth of CAP257 plasma was mediated by at least three distinct antibody specificities.

To identify the targets of each of the three broadly neutralizing antibody specificities in CAP257 plasma we first assessed whether they targeted epitopes in monomeric gp120 (such as the CD4bs or N301/N332 glycans). We adsorbed out the gp120 binding antibodies in plasma samples from the peak neutralizing activity of each wave using recombinant ConC gp120 coupled to tosyl-activated magnetic beads (Figure 1C). The adsorbed plasma was compared with untreated plasma for activity against a heterologous virus neutralized by each wave. Neutralization by wave 1 (67 weeks p.i.) and wave 3 (213 weeks p.i.) was not affected by adsorption with monomeric gp120 (Figure 1C – red and brown curves), however neutralization by wave 2 antibodies (122 weeks p.i.) could be partially adsorbed with the ConC gp120 protein (Figure 1C – blue curve). The neutralizing activity of wave 2 could also be equally adsorbed with a core gp120 lacking the hypervariable loops V1/V2 and V3 (Figure 1C – green curve). These data supported our hypothesis that CAP257 heterologous neutralization was mediated by more than one neutralizing antibody specificity, two of which were largely subtype C specific and targeted an epitope not present on monomeric gp120, and a third whose epitope in gp120 was more conserved across clades and did not require the hypervariable loops V1/V2 or V3.

### Wave 1 broadly neutralizing antibodies target V2

**Figure 2:**
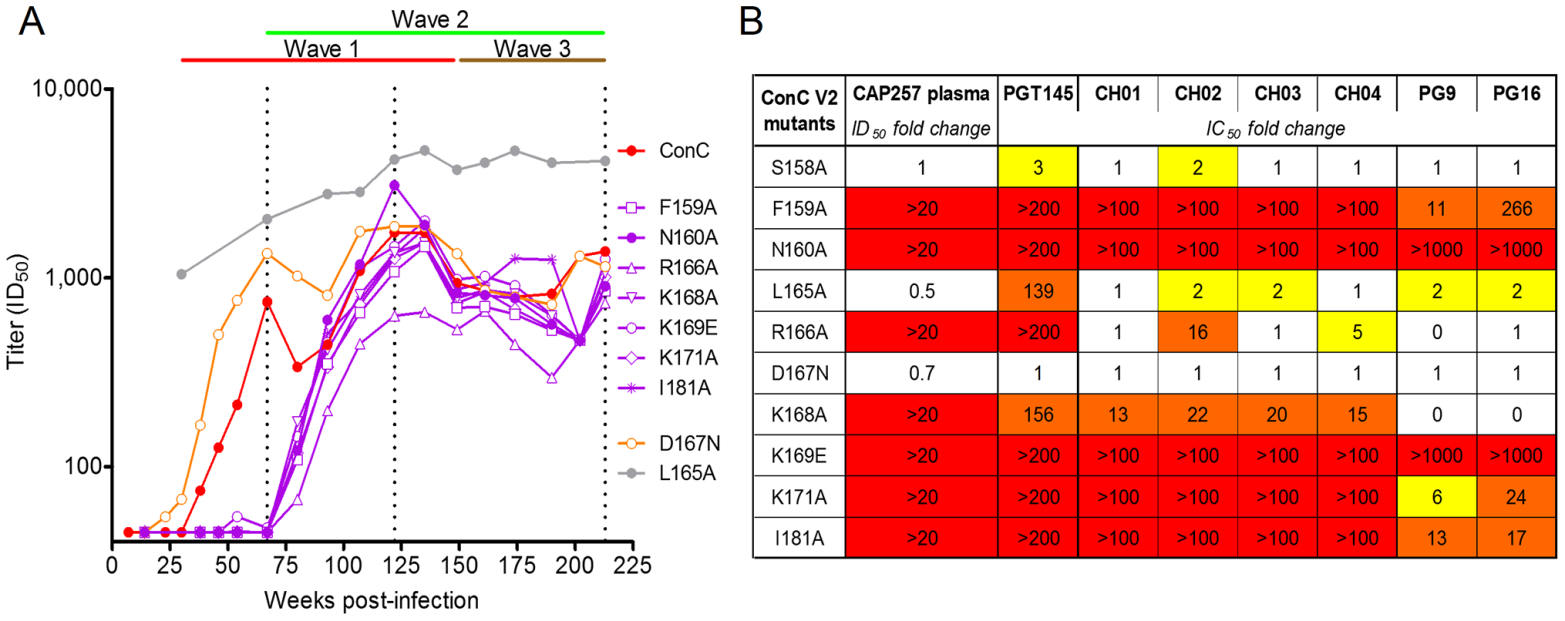
The first wave of broadly neutralizing antibodies targets residues in the V2 region. **A**) Longitudinal neutralization of ConC V2 mutants. ConC wild-type (wt) is shown in red. V2 mutants F159A, N160A, R166A, K168A, K169E, K171A, and I181A that abrogated wave 1 neutralization are shown in purple. The D167N mutation that enhanced wave 1 neutralization is shown in orange, while the L165A mutation that resulted in universal neutralization sensitivity is shown in grey. The timing of wave 1 (red), wave 2 (green), and wave 3 (brown) neutralization is summarized above as horizontal lines, while the peak titers at each wave are indicated with dotted lines. ID_50_ titers (y-axis) are shown versus weeks p.i. (x-axis). **B**) The dependence of CAP257 wave 1 neutralizing antibodies (at 67 weeks p.i.) on V2 residues in ConC, compared to monoclonal antibodies PGT145, CH01-04, and PG9/16. Complete abrogation of neutralization is colored red, 2–10 fold reductions in IC_50_ are colored yellow, and >10 fold reductions in IC_50_ are colored orange.

The inability of gp120 to adsorb out wave 1 neutralization suggested these antibodies might recognize the trimer specific epitope in V1/V2 defined by PG9/16 [16]. Therefore, we performed mapping studies using ConC, which was neutralized by all three waves of neutralizing antibodies (Figure 2A – red curve). Seven mutations in the V2 region (F159A, N160A, R166A, K168A, K169E, K171A, and I181A) each abrogated wave 1 neutralization, but did not significantly affect the titers of waves 2 or 3 (Figure 2A – purple curves). In contrast, a D167N mutation resulted in enhanced neutralization by wave 1 antibodies, but did not significantly affect the titers of waves 2 or 3 (Figure 2A – orange curve). A second mutation (L165A) also resulted in significant neutralization enhancement at all the time points tested, including those preceding breadth (Figure 2A – grey curve), suggesting that this mutation resulted in general neutralization sensitivity. Overall, these data indicated that wave 1 antibodies (but not waves 2 or 3) targeted residues in the V2 region.

To define whether the V2 epitope recognized by CAP257 plasma antibodies overlapped with that of known broadly neutralizing antibodies to this site, we tested the sensitivity of PG9/16, CH01-04, and PGT145 to the same ConC V2 mutations described above and compared them to CAP257 neutralizing antibodies at the peak of wave 1 activity, 67 weeks p.i. (Figure 2B). Of the seven mutations that abrogated CAP257 neutralization only two (N160A and K169E) resulted in complete resistance to all the antibodies tested, consistent with previous data [35], [36]. Neither the monoclonal antibodies nor CAP257 wave 1 antibodies were sensitive to deletion of the N156 glycan (through the S158A mutation) in ConC. Lastly, mutations at two hydrophobic amino acids in V2 (F159A and I181A) that do not form part of the PG9 epitope as defined by the crystal structure [37], had a significant effect on the neutralization of monoclonal antibodies targeting V2, and CAP257 wave 1 antibodies.

### Immunotype switching within V2 precedes the development of wave 1 antibodies

**Figure 3:**
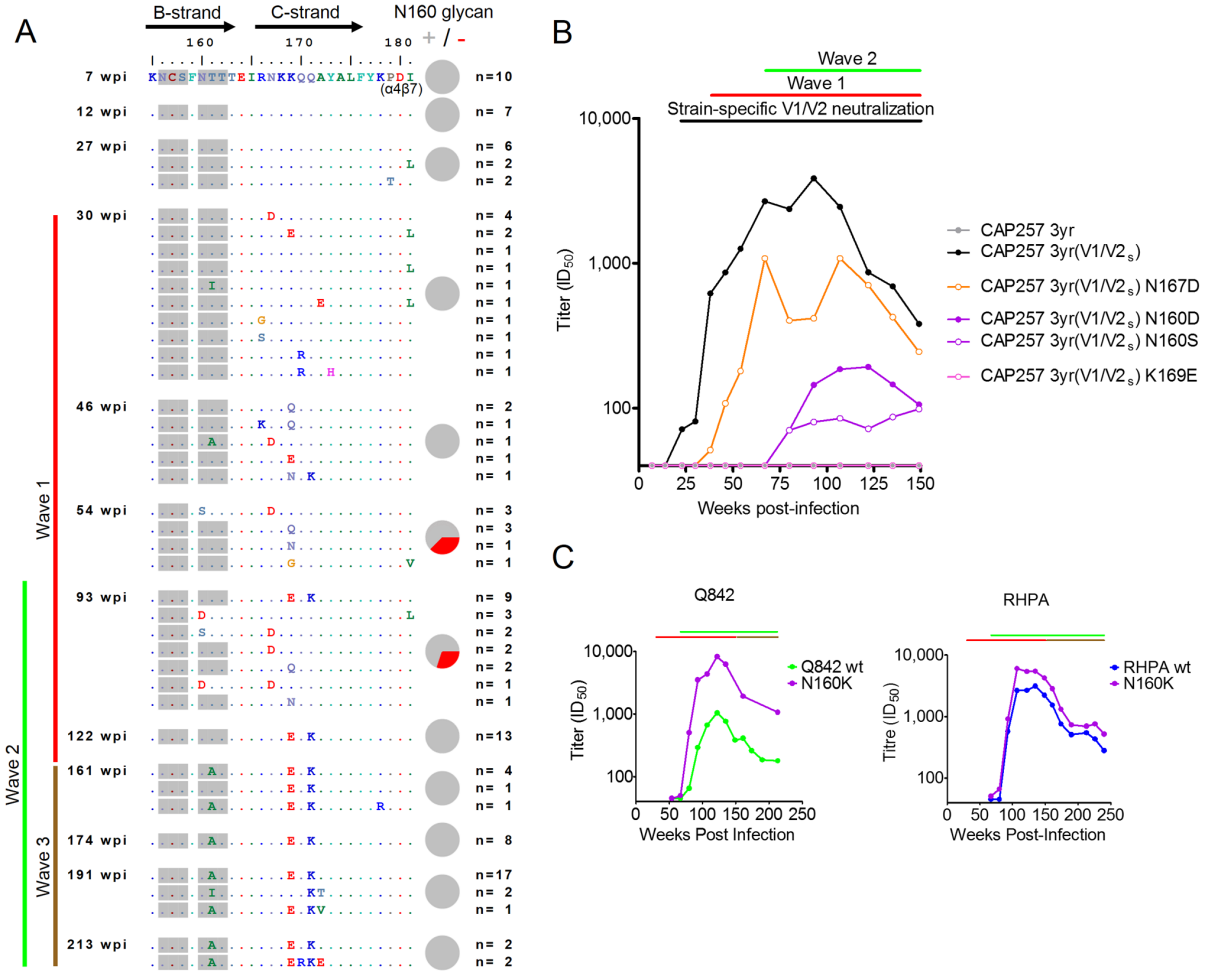
Escape from V2 neutralizing antibodies drives the formation/exposure of broadly neutralizing antibody epitopes in the CD4bs. **A**) Amino acid sequence alignment of the CAP257 B- and C- strands in the V1/V2 sub-domain of gp120, from twelve time points. The number of envelopes per unique V2 sequence is shown on the right. The timing of wave 1 (red), wave 2 (green), and wave 3 (brown) neutralization is summarized to the left with vertical lines. Potential N-linked glycans are shaded grey, and the presence (grey slices) or absence (red slices) of the N160 glycan within the population at each time point is shown with pie charts to the right. **B**) CAP257 develops a strain-specific V2 response prior to wave 1 broadly neutralizing antibodies. Neutralization of an autologous virus amplified from 174 weeks p.i. (CAP257 3 yr), is shown in grey. The V1/V2 region of this virus was back-mutated to the earliest known sequence (CAP257 3 yr(V1/V2s)) shown in black. Longitudinal neutralization of the N167D, N160D/S, and K169E mutants is shown in orange, purple, and pink respectively. The timing of wave 1 (red), wave 2 (green), and preceding strain-specific V2 (black) neutralization is summarized above with horizontal lines. ID_50_ titers (y-axis) are shown versus weeks p.i. (x-axis). **C**) Wave 2 neutralization of Q842 (green) or RHPA (blue) wild-type (wt) viruses, and their N160K mutants (purple). The timing of wave 1 (red), wave 2 (green), and wave 3 (brown) neutralization is summarized above as in Figure 1B. ID_50_ titers (y-axis) are shown versus weeks p.i. (x-axis).

To define escape from wave 1 neutralizing antibodies, we examined sequences from the V2 region of CAP257 over time. Using single genome amplification (SGA) we obtained 125 full envelope sequences from twelve time points between 7 and 213 weeks p.i., and focused on the N160 glycan and the cationic C-strand in V2 that are the targets of wave 1 antibodies (Figure 3A). Interestingly, the earliest virus (7 weeks p.i.) had an asparagine at position 167. This N167 residue is rare, occurring in only 5.6% (196 of 3,478) of sequences in the Los Alamos National Laboratory (LANL) HIV sequence database. By the time of the earliest detectable heterologous neutralization (30 weeks p.i., maximum titer of 1∶49) mutations in sites forming part of the wave 1 V2 epitope were already apparent in 6/14 autologous sequences at positions R166, K169, and Q170 (Figure 3A). Of the remaining eight sequences, six exhibited other mutations either in the N160 glycosylation sequon or the V1/V2 C-strand. This rapid selection pressure in the C-strand of V1/V2 was sometimes an N167D mutation (4/14 autologous sequences) that was unlikely to be selected for by wave 1 broadly neutralizing antibodies, as all the heterologous viruses neutralized by wave 1 had a D167 residue. Since the V1/V2 region is a common target of strain-specific neutralizing responses [38]–[41], these data suggested the possibility of an earlier neutralizing response targeting N167 in V2 that preceded the development of broadly neutralizing antibodies. Wave 1 mapping data (Figure 2A) further supported this possibility because the reverse D167N mutation enhanced the neutralization of ConC by wave 1 antibodies only, and resulted in earlier neutralization kinetics (Figure 2A – orange curve).

To test this we selected an envelope from 174 weeks p.i. (CAP257 3 yr) that was completely resistant to CAP257 neutralizing antibodies (consistent with ongoing neutralization escape), and back-mutated the V1/V2 region to match the earliest sequence from 7 weeks p.i. (Figure 3B). The neutralization sensitivity of the back-mutated virus, CAP257 3 yr(V1/V2_s_), was then compared to the parental CAP257 3 yr virus using longitudinal plasma samples. In contrast with the resistant CAP257 3 yr virus, the back-mutated V1/V2 virus (CAP257 3 yr(V1/V2s)) became sensitive to neutralization at 23 weeks p.i. (Figure 3B – black curve). This suggested the emergence of a strain-specific V1/V2 response 7 weeks prior to the development of wave 1 broadly neutralizing antibodies at 30 weeks p.i.

To establish whether these strain-specific V1/V2 neutralizing antibodies targeted the same epitope as wave 1 broadly neutralizing antibodies, we introduced selected escape mutations (N167D, N160D/S and K169E) into the sensitive CAP257 3 yr(V1/V2_s_) back-mutated envelope. Introduction of the N167D mutation (Figure 3B – orange curve), a common V2 change at 30 weeks p.i., shifted the timing of autologous V1/V2 neutralization to overlap with the emergence of wave 1 broad neutralization. The introduction of N160D/S mutations that deleted the N160 glycan (Figure 3B – purple curves), further shifted autologous neutralization to overlap with the emergence of wave 2 neutralizing antibodies. Finally, when the K169E mutation was introduced (Figure 3B – pink curve), autologous V1/V2 neutralization titers were completely abrogated. As the N160A and K169E mutations in ConC also completely abrogated neutralization by CAP257 wave 1 broadly neutralizing antibodies (Figure 2B) these data suggest that the strain-specific V2 neutralizing antibodies in CAP257 plasma targeted the same site of vulnerability that was later targeted by wave 1 broadly neutralizing antibodies. However the strain-specific V2 antibodies recognized the rare N167 immunotype of V2 present in the CAP257 infecting virus. Following an early N167D escape mutation at this site, V1/V2 neutralization became N167 independent, allowing recognition of the more common D167 immunotype. This switch in the fine specificity of CAP257 V2 antibodies correlated with the emergence of broadly neutralizing wave 1 antibodies.

### Escape from wave 1 antibodies exposed the epitope for wave 2 neutralization

Wave 1 broadly neutralizing antibodies were completely dependent on the glycan at N160 (Figure 2A). Viral escape from wave 1 neutralizing antibodies by deletion of this glycan first occurred at 54 weeks p.i. (in 37.5% of the sequences), immediately prior to the development of wave 2 neutralizing antibodies (Figure 3A – pie charts). This escape pathway persisted at 93 weeks p.i. (in 30% of sequences), but by 122 weeks p.i., at the peak of wave 2 activity, alternative escape pathways existed, and all sequences contained the N160 glycan. The transient nature of this highly effective escape pathway suggested that deletion of the N160 glycan had a deleterious effect on the virus.

A potential mechanism for this came from the observation that deleting the N160 glycan (critical to wave 1 neutralization) in the CAP257 3 yr(V1V2s) virus conferred slight neutralization sensitivity coinciding temporally with wave 2 (Figure 3B – purple curves). These data suggested that mutations at N160 exposed the wave 2 epitope. To examine whether loss of the N160 glycan enhanced CAP257 wave 2 neutralization we selected two viruses, Q842 and RHPA, which were neutralized at high titer by wave 2 but resistant to wave 1, and deleted the N160 glycan in each. The effect of these N160K mutations was assessed longitudinally using CAP257 plasma. While the timing of RHPA N160K and Q842 N160K neutralization by CAP257 was not altered compared to the wild-type viruses, these mutant viruses were neutralized 2–8 fold more potently by wave 2 antibodies (Figure 3C – purple curves). A similar 2 fold increase in titer was shown at the peak of wave 2 activity when the N160 glycan was deleted in ConC (Figure 2A – purple closed circles). As deletion of the N160 glycan in the autologous virus occurred prior to the development of wave 2 neutralizing antibodies, these data suggest that this particular escape pathway from wave 1 neutralizing antibodies may have contributed to the development of wave 2 antibodies, possibly by better exposing the epitope. Therefore, after the development of wave 2, the K169E mutation that also allowed escape from wave 1 broadly neutralizing antibodies, but did not enhance wave 2 neutralization, was preferentially selected over deletion of the N160 glycan (Figure 3A).

### Wave 2 neutralizing antibodies target the CD4bs

**Figure 4:**
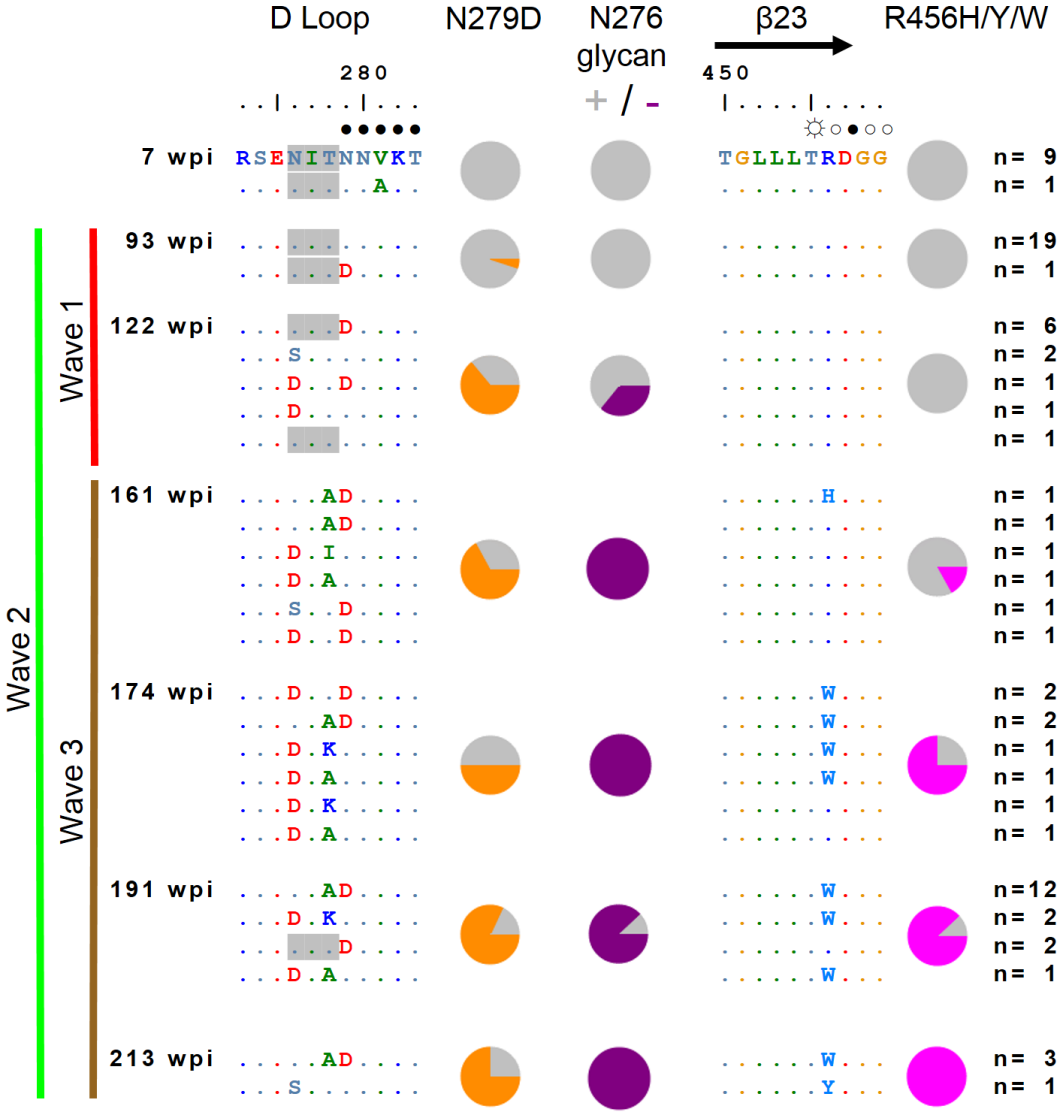
Accumulating escape mutations from wave 2 broadly neutralizing antibodies occur in the CD4 binding site. Amino acid sequence alignment of the CAP257 D loop and β23 regions of gp120 from seven time points. The number of envelopes per unique sequence is shown on the right. The timing of wave 1 (red), wave 2 (green), and wave 3 (brown) neutralization is summarized to the left with vertical lines. Amino acids contacting CD4 (as described in [57]) are indicated above the sequence alignment, with ○ denoting backbone only contacts, 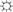 denoting side chain contacts, and • denoting main chain and side chain contacts. Potential N-linked glycans are shaded grey. The frequency of escape mutations at position 279 (orange slices), in the N276/T278 glycosylation sequon (purple slices), or at position 456 (pink slices) for each time point is shown with pie charts.

**Figure 5:**
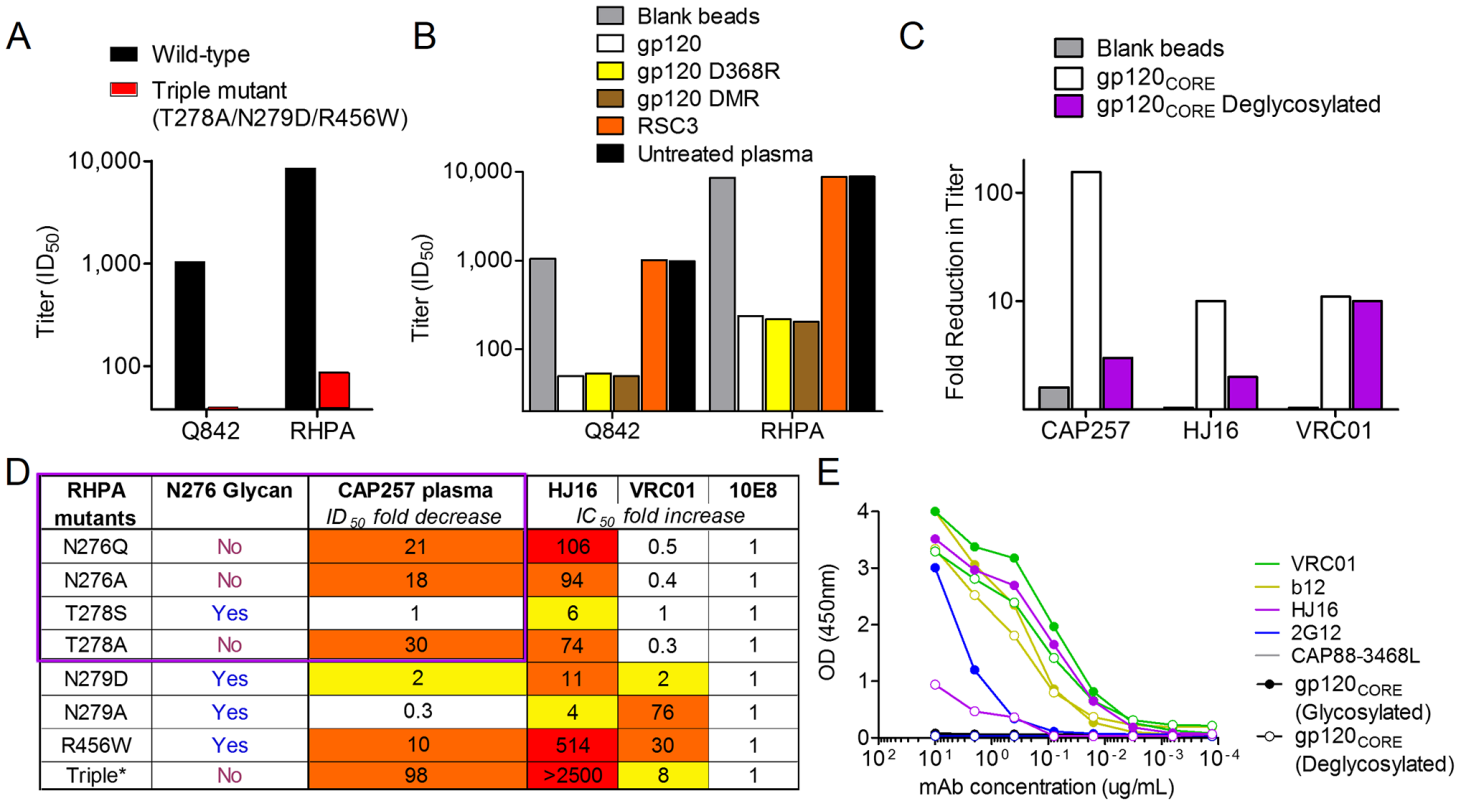
CAP257 broadly neutralizing antibodies bind a glycan dependent epitope in the CD4bs, also targeted by mAb HJ16. **A**) Effect of escape mutations T278A, N279D, and R456W in the CD4bs on wave 2 neutralization. ID_50_ titers at 122 weeks p.i. (y-axis) are shown for Q842 and RHPA (black bars), or the corresponding triple mutants (red bars). **B**) Adsorption of peak wave 2 titers (122 weeks p.i.) with wild-type or mutant gp120 proteins. The residual neutralizing activity in the adsorbed plasma samples is shown as ID_50_ titers (y-axis) for the heterologous viruses Q842 and RHPA. Untreated plasma is shown in black, plasma adsorbed with blank beads in grey, plasma adsorbed with ConC gp120 in white, plasma adsorbed with D368R mutant gp120 in yellow, plasma adsorbed with D474A/M475A/R476A triple mutant gp120 in brown, and plasma adsorbed with RSC3 protein in orange. **C**) Effect of glycosylation on the CAP257 wave 2 epitope in RHPA. The fold reduction in titer (y-axis) of adsorbed samples from 122 weeks p.i. are shown for CAP257 wave 2, HJ16, and VRC01. Plasma adsorbed with blank beads is shown in grey, gp120 core in white, and deglycosylated gp120 core in purple. **D**) The dependence of CAP257 wave 2 neutralizing antibodies (at 122 weeks p.i.) on D-loop and β23 residues/glycans in RHPA, compared to the monoclonal antibodies HJ16, VRC01, and 10E8. The effect of N276 glycan mutations on CAP257 plasma is boxed in purple, and the presence or absence of a glycan at N276 for each mutation is indicated. Fold effects between 2–10 are yellow, 10–100 colored orange, and >100 colored red. Triple* is the triple mutant described in A. **E**) Effect of glycosylation on the HJ16 epitope. The ELISA OD at 450 nm is shown (y-axis) versus antibody concentration (x-axis). Binding to glycosylated or deglycosylated gp120 core is shown with solid or open circles respectively. The monoclonal antibodies tested were VRC01 (green), b12 (yellow), HJ16 (purple), 2G12 (blue), and CAP88-3468L (grey).

The V1/V2 sub-domain of gp120 plays an important role in shielding the envelope from neutralization. More specifically, several modifications in V1/V2 (particularly at N-glycosylation sites) have been implicated in either shielding or exposing the CD4bs to neutralizing antibodies [42]–[51]. As escape from wave 1 at N160 enhanced neutralization by wave 2, these data suggested the CD4bs as the target for wave 2 antibodies. This was supported by the adsorption data (Figure 1C), showing that wave 2 neutralizing antibodies bound the core gp120 protein. Therefore we examined envelope sequences from 191 weeks p.i. for selection pressure in the conserved CD4bs (Figure 4). Three mutations in the D-loop (N276D/S, T278A/K and N279D) and one at the base of V5 in the β23 sheet of C4 (R456W) dominated the viral population at this time point. Both the D-loop and V5 have been previously implicated in resistance to CD4bs antibodies [52]–[54]. To assess whether these CD4bs mutations mediated resistance to wave 2 neutralization, we introduced the three most common mutations (T278A, N279D and R456W) simultaneously into two heterologous viruses (Q842 and RHPA) neutralized by wave 2 but not by wave 1 (Figure 1A), and tested them against plasma from the peak of wave 2 activity (122 weeks p.i.). The mutants were at least 20 fold more resistant to neutralization at this time point than the wild-type viruses, confirming the role of CD4bs mutations in escape from wave 2 antibodies (Figure 5A).

To further characterize the epitope targeted by wave 2 antibodies, we assessed the dependence of wave 2 binding on the D368 residue in α3 (critical for VRC01-like antibodies), and residues 474/475/476 in α5 (critical for HJ16-like antibodies) by adsorption studies. The D368R or D474A/M475A/R476A mutations were separately introduced into ConC gp120, and compared to the wild-type protein for their ability to adsorb out the neutralizing activity against Q842 and RHPA at peak wave 2 titers. Both mutant gp120s (Figure 5B – yellow and brown bars) adsorbed out a significant fraction of the neutralizing activity against Q842 and RHPA, equivalent to that adsorbed by wild-type gp120 (Figure 5B – white bars). These data suggested that CAP257 antibody binding was not dependent on these residues in the α3 and α5 helices. CAP257 neutralization could not be adsorbed with the RSC3 protein used to isolate VRC01, which binds weakly to the HJ16 class of CD4bs antibodies [55].

### CAP257 wave 2 antibodies recognize a glycan dependent epitope in the CD4bs, also recognized by the monoclonal antibody HJ16

Of the three changes identified above as mediating escape from wave 2 antibodies, N279 and R456 make contact with CD4, while T278 forms part of an adjacent glycosylation sequon [6]. This glycosylation sequon is conserved in 96% (n = 3,475) of envelope sequences in the LANL HIV sequence database. Its deletion via N276D/S or T278A/K mutations in later viruses (Figure 4) for wave 2 escape was therefore striking, and suggested a possible role for glycan recognition by CAP257 wave 2 antibodies. To examine wave 2 glycan binding, we expressed an RHPA core gp120 in GnTI(−/−) 293S cells, which allowed for deglycosylation of the protein using Endo-H, and assessed the ability of both glycosylated and deglycosylated proteins to adsorb out wave 2 neutralization activity. While neutralizing activity against RHPA was efficiently adsorbed with the glycosylated RHPA gp120 core (Figure 5C – white bars), the deglycosylated protein only adsorbed out a fraction of that activity (Figure 5C – purple bars) confirming the importance of glycans in wave 2 antibody binding. Similarly HJ16 was not adsorbed by the deglycosylated protein suggesting glycan dependence. In contrast, VRC01 was adsorbed equally effectively by both proteins.

To assess in more detail the role of the N276 glycan in wave 2 neutralization, we generated four mutants in RHPA, comparing the effects of two conservative mutations (N276Q, T278S) with the effects of two alanine substitutions (N276A, T278A) in the N276 glycosylation sequon (Figure 5D – boxed in purple). Both alanine mutations significantly affected wave 2 peak titers by 18 and 30 fold respectively. The N276Q mutation which deleted the glycan but retained the amino acid properties at position 276 also affected wave 2 neutralization by 21 fold (a similar effect to the N276A mutation), while the T278S mutation that retained the N276 glycan had no effect on neutralization. These data suggested that sensitivity to wave 2 neutralization was largely dependent on the glycan at position 276, rather than the N276 amino acid side chain. We also assessed the effect of the remaining two autologous mutations in the CD4bs (N279D and R456W) identified above (Figure 5D). The N279D mutation alone had a relatively small 2 fold effect on neutralization, suggesting only a minor role in escape from wave 2. When an alanine was substituted at position 279 instead, wave 2 neutralization was enhanced. The R456W mutation had a more significant 10 fold effect on CAP257 neutralization, but was still less effective than the glycan deleting mutations at positions 276/278 which were the major escape mutations.

We next compared the epitope for CAP257 wave 2 neutralizing antibodies with that of HJ16 (CD4bs/DMR) and VRC01 (CD4bs). Both monoclonal antibodies were profoundly affected by the R456W mutation (514 and 30 fold respectively). Like CAP257, HJ16 neutralization was significantly dependent on the glycan at position 276 with glycan deleting mutations (N276Q/A and T278A) resulting in a 74–106 fold increase in IC_50_ (Figure 5D). This dependence on the N276 glycan distinguished both CAP257 and HJ16 from VRC01, for which neutralization was slightly enhanced (2–3 fold) when the glycan was removed. This effect on VRC01 is consistent with previous studies showing that deleting the N276 glycan exposes the CD4 binding site to neutralization by VRC01 or b12 [52], [56]. Introducing all three CAP257 escape mutations identified above therefore had a compensatory effect on VRC01 resistance (8 fold compared to 30 fold effect for R456W alone), but completely abrogated HJ16 neutralization, confirming the similarities between HJ16 and CAP257 plasma. Despite these overall similarities, some differences were apparent between CAP257 wave 2 antibodies and HJ16, such as the preference of HJ16 for the threonine at position 278 and the asparagine at position 279. Unlike CAP257, the N279A mutation did not enhance HJ16 neutralization but rather resulted in a 4 fold reduction in titer. These data may suggest minor contacts between HJ16 and the amino acid side chain at position 279.The N279A mutation also significantly affected VRC01 neutralization (76 fold), consistent with either asparagine or aspartic acid residues at position 279 being directly contacted by W100^B^ in the CDR3-H3 of VRC01 [52], [57].

To clarify the role of glycan binding we tested three CD4bs neutralizing antibodies (VRC01, b12, and HJ16) in ELISA for binding to either the glycosylated or deglycosylated RHPA gp120 core proteins (Figure 5E). The neutralizing antibody 2G12 has a well-defined glycan epitope and was used as a positive control [58]–[62], while CAP88-3468L (a V3 binding antibody) served as a negative control [63]. Deglycosylation significantly affected the binding of both 2G12 and HJ16 (Figure 5E – blue and purple curves), but did not significantly affect binding of either VRC01 or b12 (Figure 5E – green and yellow curves) to the RHPA gp120 core. These data confirmed the glycan binding properties of HJ16, and suggest that both CAP257 wave 2 neutralizing antibodies and HJ16 have a glycan dependent mechanism of neutralization at the CD4bs.

### Early wave 2 escape mutations drive an increase in neutralization breadth

While the simultaneous introduction of T278A, N279D, and R456W mutations into heterologous viruses Q842 and RHPA made them resistant to wave 2 neutralization at 122 weeks p.i. (Figure 5A), when each mutation was introduced individually into RHPA the results were varied (2–30 fold reductions in titer) with no single mutation resulting in complete escape (Figure 5D). These data suggested that escape from wave 2 required a combination of all three mutations. Longitudinal sequence analysis showed that the N279D mutation emerged first at 93 weeks in 5% of the population, followed by N276D/S glycan deleting mutations at 122 weeks in 36% of the population, and then by substitution of the R456 residue with a bulky amino acid side chain (H, Y, or W) at 161 weeks p.i. in 17% of the population (Figure 4).

**Figure 6:**
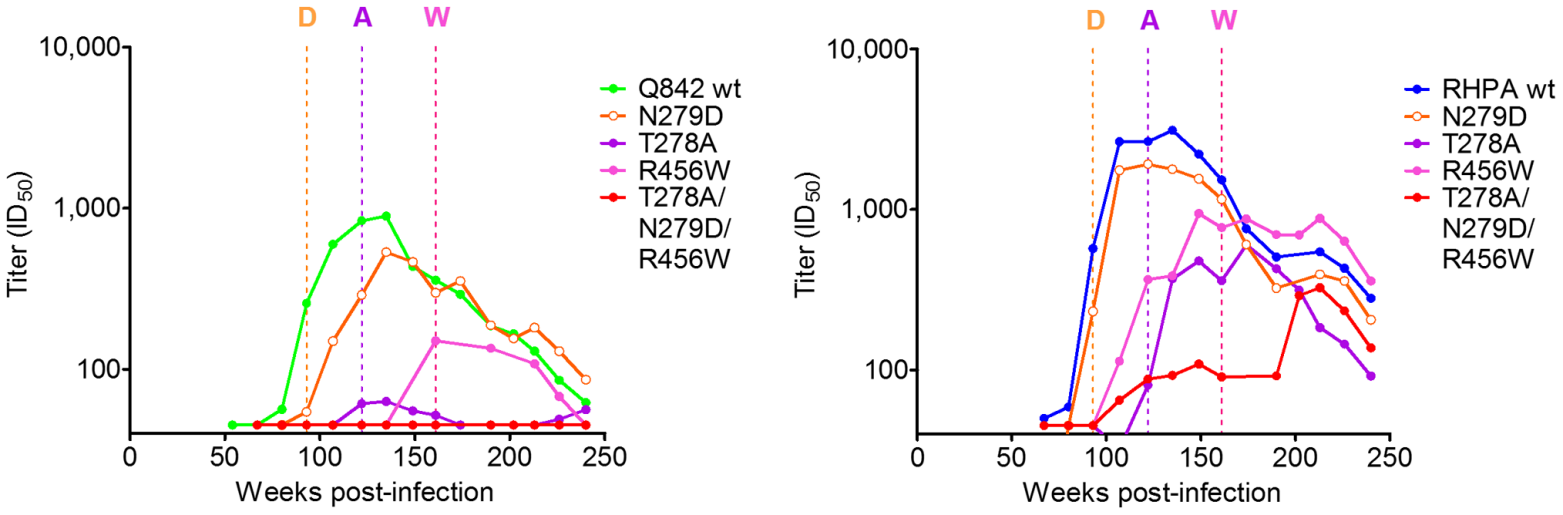
Changes in the fine specificity of CAP257 CD4bs antibodies in response to autologous escape mutations. The longitudinal neutralization of Q842 (green) or RHPA (blue) wild-type (wt) viruses was compared to the neutralization of N279D (orange), T278A (purple), and R456W (pink) mutant viruses. The triple mutant (T278A/N279D/R456W) from Figure 5A is also shown in red. ID_50_ titers (y-axis) are shown versus weeks p.i. (x-axis). Dotted lines indicate the time points at which each mutation (D = N279D, A = T278A, W = R456W) first appears in the autologous sequences (colored as above).

To better characterize their contributions to escape from wave 2 antibodies, each mutation (T278A, N279D, and R456W) was introduced separately into Q842 and RHPA and compared to the wild-type viruses. The N279D mutation (Figure 6 – orange curves) had a significant effect only at the beginning of wave 2 activity against Q842, shifting the earliest heterologous neutralization of Q842 from 80 weeks to 93 weeks p.i. (with a 5 fold effect on titers at 107 weeks p.i.). The mutation also affected the titers against RHPA by 2–3 fold. This suggested an initial dependence on N279, with later wave 2 antibodies being less vulnerable to mutations at this residue. This reduced dependence on N279 coincided with the emergence of the N279D mutation at 93 weeks p.i. (Figure 6 – orange dotted lines) providing a mechanism for maturation of the antibody response. The T278A mutation (Figure 6 – purple curves) that deleted the N276 glycan conferred almost complete resistance in Q842, and shifted the earliest neutralization of RHPA from 67 to 122 weeks p.i. The R456W mutation (Figure 6 – pink curves) did not result in a further right shift in the neutralization curves for RHPA relative to the T278A mutation, but did affect the neutralization kinetics of Q842 relative to the wild-type or N279D mutant viruses. As with the N279D mutation, these changes in the fine specificity of the maturing antibody response reflected the emergence of either T278A or R456W mutations in longitudinal autologous sequences (Figure 6 – purple and pink dotted lines respectively).

**Figure 7:**
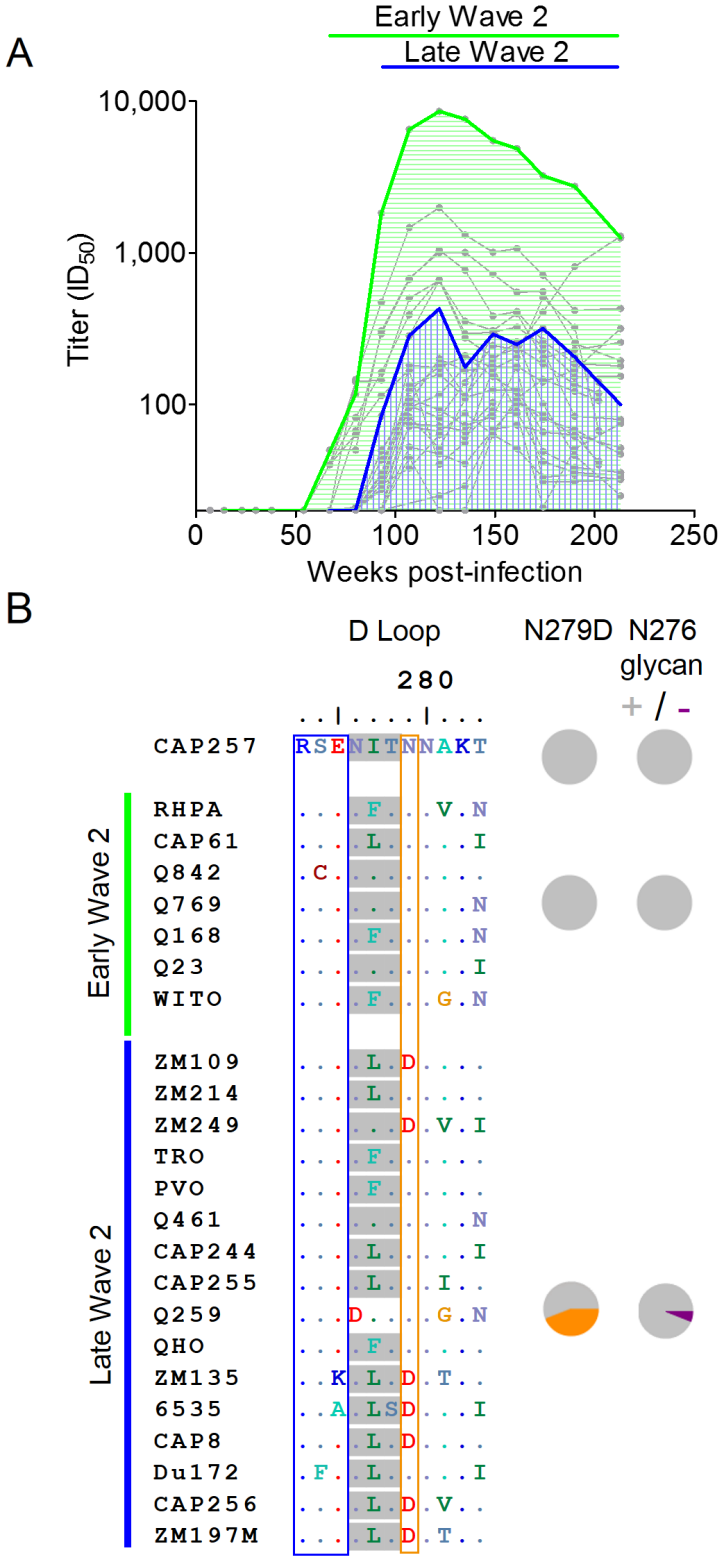
Maturation of the wave 2 CD4bs response results in the increased neutralization breadth of CAP257 plasma. **A**) Summary of early (green) and late (blue) heterologous neutralization by wave 2 antibodies superimposed over the individual virus neutralization kinetics (grey). Viruses neutralized by V2 antibodies in wave 1 have been excluded. ID_50_ titers (y-axis) are shown versus weeks p.i. (x-axis). **B**) Amino acid sequence alignment of heterologous viruses depicted in (A). The timing of early or late heterologous neutralization is shown on the left with horizontal lines. The N276 glycan is shaded grey, and position 279 is boxed in orange. The frequency of the N279D mutation or disrupted N276 glycosylation within the two groups is shown with orange or purple pie slices respectively. The N-terminal region of the D-loop is boxed in blue.

These data are consistent with accumulating resistance to wave 2, and suggest that after each successive round of escape, new antibody variants emerged that were able to neutralize first the N279D mutant, and then later autologous viruses with additional polymorphisms at positions 276, 278, or 456. We wished to determine whether the ability to neutralize escaped viral variants correlated with increased wave 2 neutralization breadth. Heterologous viruses neutralized by wave 2 were divided into two groups, those neutralized at 67 weeks p.i. (early wave 2 neutralization), and those first neutralized at 93 weeks p.i. (late wave 2 neutralization) after the emergence of initial wave 2 escape mutations (Figure 7A). Viruses also neutralized by wave 1 were omitted as the overlapping titers confounded this analysis. Inspection of the envelope sequences (particularly in the D-loop) showed that all of the viruses neutralized by early wave 2 antibodies had the N279 immunotype (Figure 7B – boxed in orange). In contrast 44% of viruses neutralized by later wave 2 antibodies had the D279 immunotype. Furthermore, of the viruses neutralized by later wave 2 antibodies, one (Q259) lacked the N276 glycan and four others also had additional non-conservative mutations at positions 273–275 in the N-terminus of the D-loop (Figure 7B – boxed in blue) that may also affect early wave 2 neutralization. These data suggest that wave 2 escape mutations guided maturation of the CD4bs response, enabling later wave 2 antibodies to neutralize additional heterologous viruses and ultimately resulting in the increased neutralization breadth of CAP257 plasma.

### The wave 3 neutralizing antibody target is distinct but undefined

**Figure 8:**
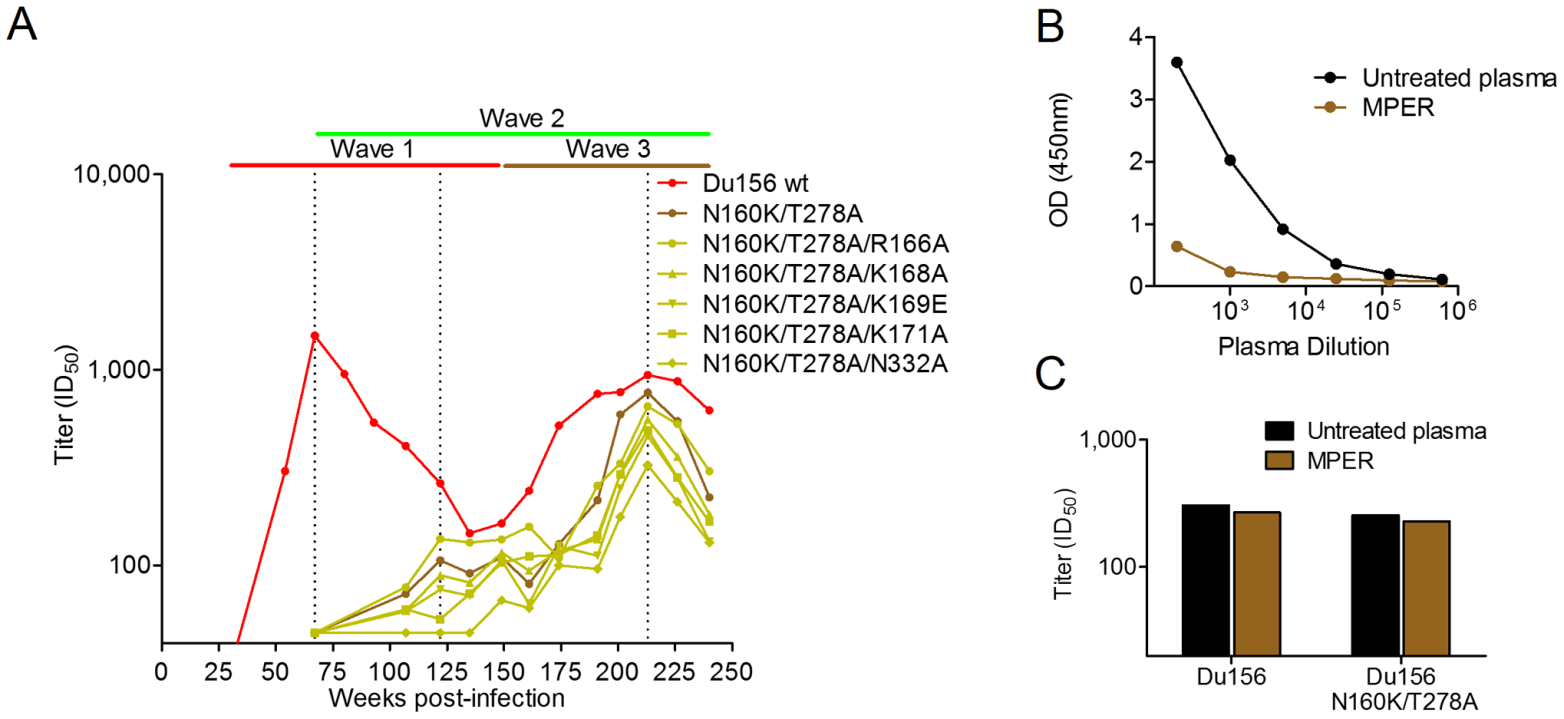
Wave 3 neutralizing antibodies target a novel epitope. **A**) Neutralization of Du156 wild-type (wt) virus by longitudinal CAP257 serum is shown in red. The timing of wave 1 (red), wave 2 (green), and wave 3 (brown) neutralization is summarized above as horizontal lines, with peak titers of each wave indicated with dotted lines. The Du156 N160K/T278A double mutant is shown in brown. Du156 triple mutants N160K/T278A/(R166A, K168A, K169E, K171A, or N332A) are shown in yellow. ID_50_ titers (y-axis) are shown versus weeks p.i. (x-axis). **B**) Adsorption of MPER binding antibodies. OD (450 nm) against the MPER peptide is shown (y-axis) versus plasma dilution (x-axis). Untreated plasma is shown in black, and MPER adsorbed plasma in brown. **C**) Adsorption of wave 3 neutralization by MPER (colored as above). ID_50_ neutralization titers at 213 weeks p.i. (y-axis) are shown for Du156 and Du156 N160K/T278A.

Like wave 1, wave 3 neutralization could not be adsorbed with monomeric gp120, suggesting that these antibodies targeted a quaternary epitope, or an epitope in gp41. To assess whether wave 3 was a distinct antibody specificity, or a re-emergence of wave 1 antibodies, we selected a virus (Du156) that was sensitive to waves 1 and 3, and less sensitive to wave 2 (Figure 8A – red curve). Resistance to wave 1 (V2) and wave 2 (CD4bs) neutralizing antibodies was established by introducing the N160K and T278A mutations identified above. The resulting virus (Du156 N160K/T278A) remained sensitive to wave 3 neutralization only (Figure 8A – brown curve), suggesting that wave 3 differed from wave 1 (which was completely abrogated by the N160K mutation). To confirm this, we introduced additional V2 mutations (R166A, K168A, K169E, K171A) known to abolish wave 1 neutralization, into the Du156 double mutant. None of these mutations significantly affected the titers of wave 3 neutralizing antibodies (Figure 8A – yellow curves), confirming that the epitope for wave 3 antibodies did not overlap with the wave 1 V2 epitope. An N332A mutation was also introduced to confirm that wave 3 antibodies did not target this glycan.

To test whether CAP257 wave 3 neutralizing antibodies targeted the MPER region of gp41, we coupled MPER peptides to magnetic beads and used them to adsorb out MPER specific binding antibodies in CAP257 plasma. The adsorption of MPER binding antibodies (Figure 8B) did not affect neutralization of Du156 or the double mutant Du156 N160K/T278A (with wave 1 and 2 resistance mutations) when compared to untreated plasma (Figure 8C). Thus, while we cannot exclude the possibility that wave 3 antibodies target V2 or gp41, these neutralizing antibodies appear to target an epitope distinct from any of the four known sites of vulnerability in the HIV-1 envelope.

### CAP257 viruses develop resistance to known broadly neutralizing antibodies

**Figure 9:**
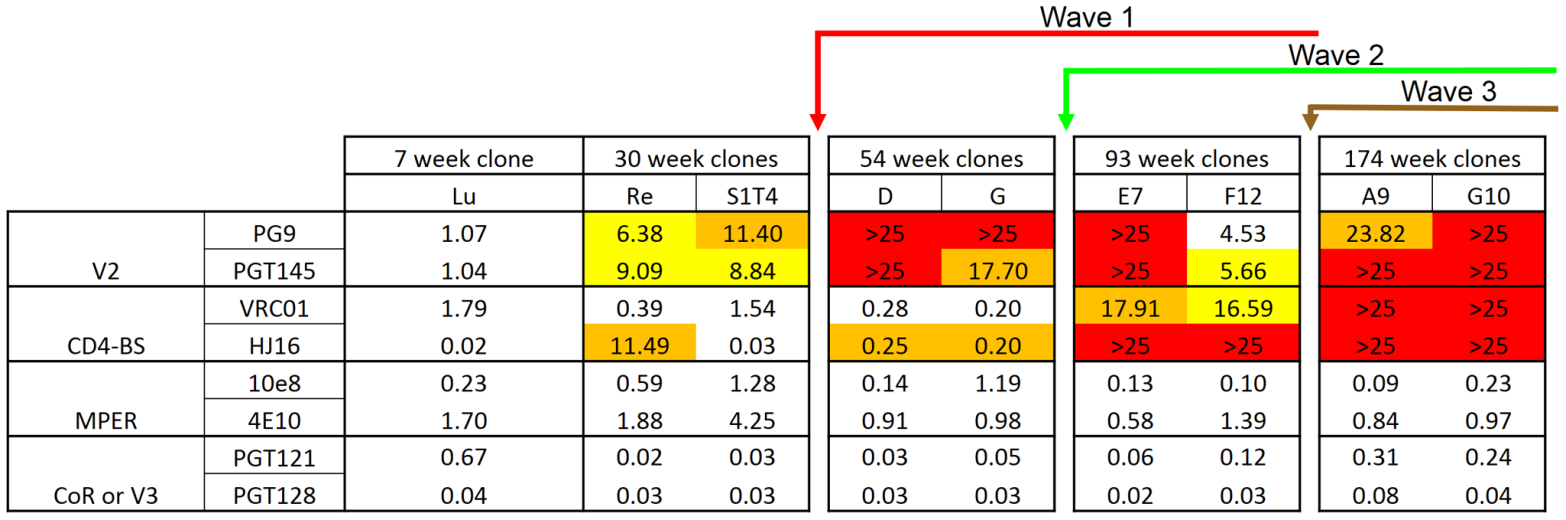
Accumulating resistance of CAP257 clones to broadly neutralizing monoclonal antibodies targeting V2 and the CD4bs. The sensitivity of nine CAP257 clones from 7, 30, 54, 93, and 174 weeks p.i. to broadly neutralizing antibodies targeting the four known sites of vulnerability was measured in a TZM-bl assay. The timing of wave 1 (red), wave 2 (green), and wave 3 (brown) neutralization is summarized above with arrows. Increases in IC_50_ relative to the 7 week clone are colored as follows: 5–10 fold (yellow), >10 fold (orange), complete neutralization resistance (red).

We hypothesized that the selection pressure exerted by these broadly neutralizing antibodies would impact on the overall neutralization phenotype of CAP257 viruses over time. Therefore we tested the sensitivity of envelopes from 7, 30, 54, 93, and 174 weeks p.i. to broadly neutralizing monoclonal antibodies targeting the four major epitopes (Figure 9). The 7 week clone from CAP257 was sensitive to neutralization by anti-V2 antibodies, but following the development of wave 1 V2 neutralizing antibodies, CAP257 viruses became more resistant to PG9 and PGT145 neutralization. The 7 week clone was also highly sensitive to HJ16 (0.02 µg/mL) and VRC01 (1.79 µg/mL), however clones isolated after the emergence of wave 2 neutralization were increasingly resistant to neutralization by antibodies targeting the CD4bs. CAP257 viruses from all the time points selected were sensitive to neutralization by antibodies targeting the MPER or N301/N332, neither of which was targeted by broadly neutralizing antibodies in CAP257 plasma. These data confirm that CAP257 developed broadly neutralizing antibodies targeting the CD4bs and V2. Furthermore, the finding that CAP257 viruses remained sensitive to neutralization by antibodies targeting N301/N332 and the MPER supports our hypothesis that wave 3 antibodies target a novel epitope on the HIV-1 envelope.

## Discussion

**Figure 10:**
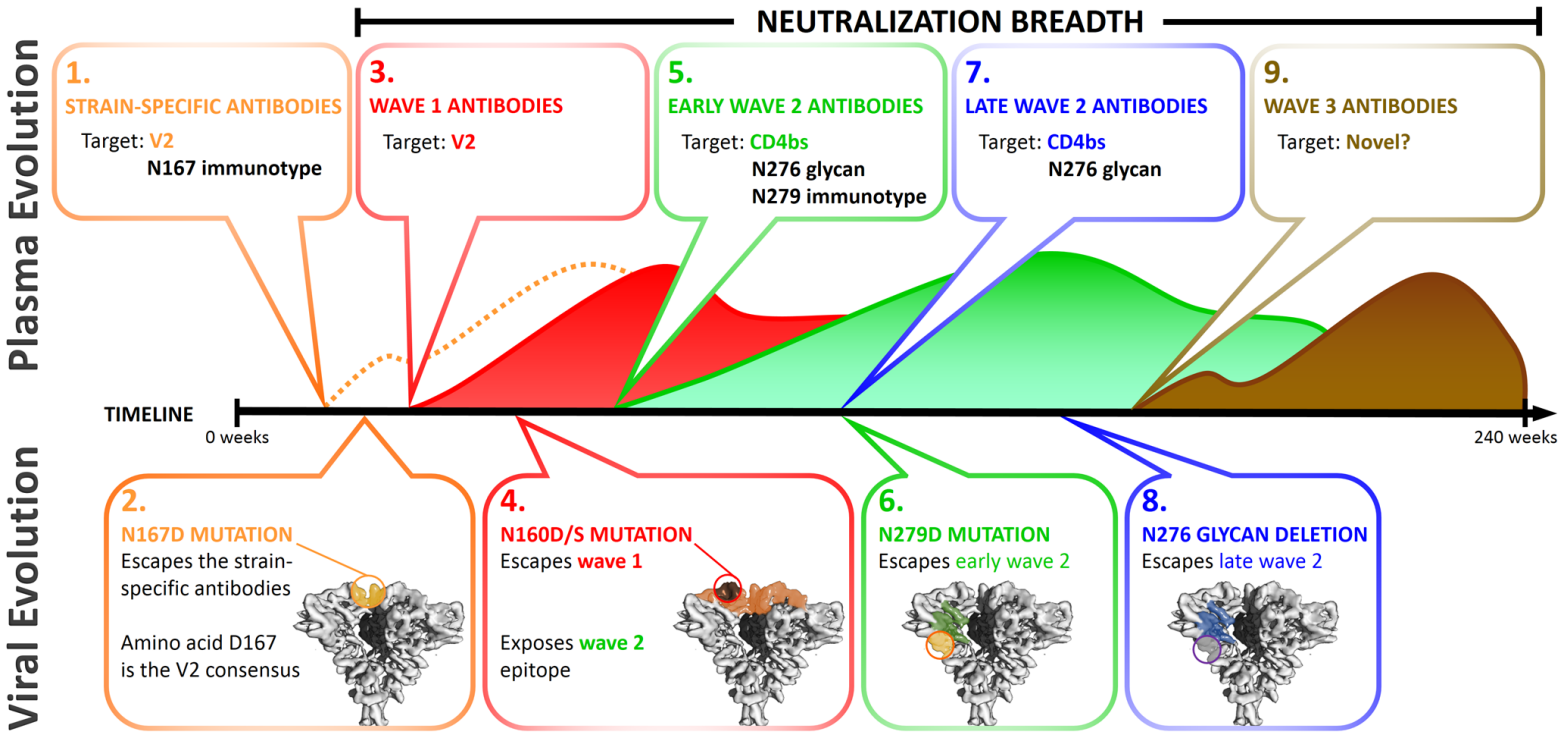
Summary of the role of CAP257 viral evolution in shaping broadly neutralizing antibody responses. The schema depicts the evolution of plasma neutralizing antibodies and viral escape mutations over 240 weeks. Each of the three waves of CAP257 broadly neutralizing antibodies is shown. Text boxes highlight the key events described herein. Env trimers (EMD-5447) were drawn in UCSF-Chimera, and spheres were used to approximate the location of escape mutations. **1**) Strain-specific antibodies (dotted orange line) developed at 23 weeks, targeting a V2 epitope overlapping with known V2 antibodies (eg: PG9). **2**) Strain-specific V2 antibodies were escaped by an N167D mutation (D being the global consensus at this site). **3**) Broadly neutralizing antibodies targeting V2 (wave 1 – red curve) developed at 30 weeks. **4**) Escape from wave 1 antibodies through deletion of the N160 glycan was associated with exposure of an epitope in the CD4bs. **5**) At 67 weeks broadly neutralizing antibodies targeting the CD4bs (wave 2 – green curve) develop. Neutralization was N276 glycan dependent and sensitive to an N279D mutation. **6**) The N279D change emerges at 93 weeks, significantly affecting wave 2 neutralization at this time point. **7**) Wave 2 neutralization becomes independent of position 279, which was associated with increased neutralization breadth. **8**) Mutations that delete the N276 glycan (as well as an R456W change) escape wave 2 antibodies. **9**) CAP257 develops a third broadly neutralizing antibody specificity that could not be mapped to any of the four known antibody targets.

A preventative HIV-1 vaccine remains the most likely way to end the HIV pandemic, but current envelope immunogens have so far failed to elicit broadly neutralizing antibodies. Nonetheless, the development of cross-reactive antibodies in approximately a quarter of HIV-1 infected individuals has confirmed that the human immune system can make such antibodies. Much emphasis has been placed on mapping the targets for these broadly neutralizing antibodies in an attempt to define viral vulnerabilities for immunogen design. Here we analyzed CAP257 heterologous neutralization over a 4.5 year period, describing the sequential evolution of three distinct broadly neutralizing antibody specificities within a single HIV-1 subtype C infected individual. We further showed how early viral evolution in the context of broadly reactive antibodies may profoundly shape the maturing antibody response towards enhanced neutralization breadth, in a process that may inform immunogen design. These data have been summarized in Figure 10.

The CAP257 autologous virus efficiently escaped all three specificities. As a consequence the antigenic stimulus for these broadly neutralizing antibodies declined, and antibody titers dropped at least ten fold within a three year period. The waxing and waning of the broadly neutralizing specificities in CAP257 confounded our previous attempts to map the targets at 174 weeks p.i. [7], when the titers of the three waves overlapped significantly. As most mapping studies are cross-sectional, the number of individuals who mount multiple broadly neutralizing antibody responses may therefore be underestimated, and may make up a significant proportion of those plasma samples that remain undefined.

Nonetheless we were able to finely map 2 of the 3 specificities in this study and showed that they targeted known sites of vulnerability on the HIV-1 envelope. Wave 1 antibodies targeted the site defined by PG9/16 and were completely dependent on N160 and K169, consistent with previous data describing PG9/16 dependence on the N160 glycan and the positively charged amino acids in the C-strand of V1/V2 [16], [37]. In general the epitope for wave 1 antibodies showed a larger footprint in V2 when compared to the epitopes of other monoclonal antibodies targeting this site, but behaved most similarly to PGT145. However none of the antibodies or plasma tested was sensitive to removal of the N156 glycan. While the crystal structure of PG9 with V2 showed interactions with the N156 glycan [37], the effect of deleting the N156 glycan is variable [16], [35], [36]. This effect might be explained by recent data suggesting that PG9 recognizes two N160 glycans (from two adjacent gp120 monomers) but only one N156 glycan [64]. The requirement for a lysine at position 169 explains the subtype C specificity of CAP257 wave 1 antibodies, as this residue is less common in subtypes A and B [35].

Wave 2 antibodies targeted a known site of vulnerability, the CD4bs, but these antibodies had an unusual glycan dependent mechanism of neutralization. CAP257 wave 2 and HJ16 neutralization were both highly dependent on interactions with the N276 glycan. N276 is also the recently described target of the broadly neutralizing antibody 8ANC195, but this antibody does not appear to interact with the CD4bs [65]. While glycan dependence for neutralization has not previously been described for CD4bs antibodies, including HJ16, Balla-Jhagjhoorsingh *et al*. reported that resistance to HJ16 involved an N276D mutation (deleting the glycan) with a hundred fold drop in titer [66], providing support for our observations. The glycan dependence of both HJ16 and CAP257 wave 2 antibodies suggests that they target a similar sub-epitope of the CD4bs that may be better defined as an N276 glycan dependent class of neutralizing antibodies, which is distinct from the VRC01 class. Recently it was shown that related variants of VRC01 do bind the glycan at N276 [67], however this glycan is not a major determinant of neutralization sensitivity to VRC01 [52], [56], [57], [68]. Rather, N276 has been described as a protective shield for the CD4bs, and deleting this glycan enhances the neutralization of CD4bs antibodies VRC01 and b12 [52], [56]. Removing N276 from gp120 also enabled binding to the predicted germline antibody for VRC01, which otherwise did not bind to gp120, and has been suggested as a modification for candidate vaccine immunogens [69], [70]. However, the glycan shield is increasingly recognized as a major site of vulnerability on the HIV-1 envelope [15], [16], and as with the glycans at N156/N160 and N301/N332, conservation of the N276 glycan bordering the CD4bs may make it a promising target for vaccine design.

**Figure 11:**
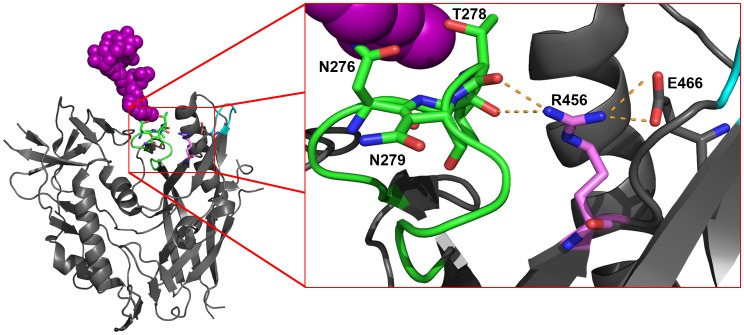
The R456 side chain stabilizes the CD4bs epitope through hydrogen bonding. A diagram of the 93TH057 gp120 crystal structure (pdb file 4JKP) shown in an orientation similar to the angle of approach for CD4. The crystalized part of the N276 attached glycan (GlcNAc_2_Man_4_) is shown with purple spheres. The D-loop is shown in green, the V5 loop is shown in cyan, and the R456 residue is shown in pink. Oxygen atoms are colored red, and nitrogen atoms blue. The inset shows a magnified view of the interaction between R456 and residues in the D-loop or the β24 strand. Putative hydrogen bonds are shown with dotted orange lines. The image was created using The PyMOL Molecular Graphics System, Version 1.3r1edu, Schrödinger LLC.

Characterization of viral escape from CAP257 CD4bs antibodies indicated that deletion of the N276 glycan alone did not confer complete resistance. Escape required accumulating mutations in the CD4bs site, consistent with the functional conservation of this epitope. In addition to deletion of the N276 glycan, CAP257 escape occurred through a R456W mutation that also significantly affected neutralization by VRC01 (30 fold) and HJ16 (514 fold). This mutation likely contributed towards the evolution of VRC01 resistant virus by 174 weeks. W456 is extremely rare, occurring in only 0.78% (27 of 3,481) of sequences in the LANL HIV-1 sequence database. A crystal complex for HJ16 is not available, however the structure of VRC01 bound to its epitope showed that this antibody does not make significant contact with the R456 side chain in gp120, but rather hydrogen bonds with the R456 backbone carbonyl group. This suggests that the R456W mutation provides an indirect mechanism for resistance. The highly conserved R456 side chain can make hydrogen bonds with backbone carbonyl groups of amino acids at position 277 and 278 in the D-loop, as well as hydrogen bonds with E466 side chain in β24, C-terminal to V5 (Figure 11). Loss of these bonds and localized conformational changes to accommodate a bulky tryptophan residue may destabilize this critical component of the CD4bs epitope [52], [56], [71].

This study adds to data showing that the immune system can target multiple conserved epitopes [29]–[31]. It is striking that in three of these four studies, antibodies targeted both V2 and the CD4bs (donors CH219, AC053, and CAP257), suggesting an association between these two epitopes. Indeed, there is a well-documented relationship between V1/V2 and the CD4bs. The V1/V2 region protects the receptor binding sites from neutralization [42]–[51], and also interacts with V3 at the trimerization domain to hold the CD4bs in its pre-liganded conformation [21]. The crystal structures of monoclonal antibodies PG9, CH58, and CH59 bound to their epitopes in V2 show that the conformation of the V1/V2 sub-domain may vary significantly, but the factors that govern these conformational states are not known [37], [72]. In CAP257, deletion of the N160 glycan increased exposure of the CD4bs. It is possible that certain immunotypes of the V1/V2 epitope, such as the rare mutations at N160 or D167 described here, shifted the equilibrium of V1/V2 toward conformations that better exposed the CD4bs to neutralizing antibodies. This is supported by previous observations that introduction of the N160K and D167N mutations simultaneously into JR-FL resulted in a >50 fold increase in neutralization sensitivity to CD4bs antibodies [73]. CAP257 was infected with the less common N167 variant, and therefore following escape from V2 wave 1 antibodies a similar conformational state (D/S160, N167) would have been presented to the immune system. Our data suggest that this escape pattern further exposed the glycan dependent CD4bs sub-epitope. Although vaccination with the K160 and/or N167 immunotype may improve antibody responses to the CD4bs site, antibodies induced to the V2 epitope would be relatively strain-specific, like monoclonal antibody 2909 that recognizes the K160 immunotype and is therefore specific for SF162 [74]. In CAP257, switching from N167 to the more common D167 residue resulted in escape from the strain-specific response to V2, and this coincided with the development of a much broader response targeting the same epitope.

Sequential immunization may be a useful strategy to promote the broadening of the B-cell response. Recently Murphy *et al.* showed that two light chain variants paired with a single heavy chain of a strain-specific neutralizing antibody differentially neutralized early autologous envelopes [75]. While evolution of that strain-specific epitope did not affect the development of broadly neutralizing antibodies in this individual, the data supports the possibility that viral evolution might facilitate the neutralization of amino acid variants within a given epitope. Similarly, we have previously shown in an individual who developed broadly neutralizing antibodies to the V2 region that viral escape drove a maturation of the antibody response towards recognizing multiple V2 variants [35]. Here we show that emergence of an aspartic acid at position 279 preceded a broadening of the B-cell response to the CD4bs. The N279 and D279 amino acid variants of the CD4bs are equally common among sequences in the LANL HIV-1 database (50% and 48% respectively, n = 3,479), and preferential neutralization of either immunotype would halve the neutralization breadth of an antibody. CAP257 wave 2 neutralizing antibodies and HJ16 were somewhat sensitive to the N279D change. However the resistance of D279 containing viruses to CAP257 antibodies was rapidly lost after the emergence of the N279D escape mutation. Therefore, like the N167D mutation in V2 described for wave 1, this change in the fine specificity of CAP257 antibodies coincided with an increase in the neutralization breadth of the CD4bs response. We hypothesize that position N279 was non-critical for antibody binding, and despite temporarily facilitating neutralization escape, the N279D mutation then promoted affinity maturation by reducing the dependence of CAP257 antibodies on this amino acid. This adds to recent data suggesting a major role for viral evolution in the development of neutralization breadth to the CD4bs [76]. These data support the possibility that a sequential immunization strategy would enhance neutralization breadth by systematically presenting common variants in a given epitope. Such residues would have to be non-critical to antibody binding allowing for the evolution of higher affinity variants that would in turn recognize multiple immunotypes.

Overall these data highlight how interactions between the host immune system and viral escape mutations shaped the development of broadly neutralizing antibodies. The escape pathways identified here that led to the increased breadth of neutralization for both V2 and CD4bs antibodies provide potential pathways for generating broadly neutralizing antibodies. Further defining these pathways through the isolation of monoclonal antibodies will provide valuable insight into how these types of antibodies could be elicited using sequential immunization in a vaccine setting.

## Materials and Methods

### Ethics statement

The CAPRISA Acute Infection study received ethical approval from the Universities of KwaZulu-Natal (E013/04), Cape Town (025/2004), and the Witwatersrand (MM040202). CAP257 provided written informed consent for study participation.

### CAPRISA 002 Acute Infection cohort participant

The CAPRISA Acute Infection cohort is comprised of women at high risk of HIV-1 infection in Kwa-Zulu Natal, South Africa [77]. Here we studied one individual (CAP257) from seven weeks p.i. through four and a half years of infection, until she started anti-retroviral therapy. During this time she had an average viral load of 60,784 copies/mL and an average CD4 count of 498 cells/µL. Plasma samples collected at 30 time points were used in this study.

### Single genome amplification

The amplification of envelope genes from single HIV-1 RNA genomes has been previously described [78]. Viral RNA was isolated from CAP257 plasma using a Viral RNA Extraction Kit (QIAGEN), and cDNA was synthesized with Superscript III Reverse Transcriptase (Invitrogen). The reaction product was treated with RNase H (Invitrogen) and envelope genes were amplified by nested PCR with Platinum Taq (Invitrogen). Amplicons were purified with a PCR Clean-up Kit (QIAGEN) and single genome amplification confirmed by DNA sequencing using the ABI PRISM Big Dye Terminator Cycle Sequencing Ready Reaction kit (Applied Biosystems) and an ABI 3100 automated genetic analyzer. The full-length *env* sequences were assembled and edited using Sequencher v.4.5 (Genecodes) and Bioedit v.7.0.5.3.

### Cell lines

The TZM-bl cell line engineered from CXCR4-positive HeLa cells to express CD4, CCR5, and a firefly luciferase reporter gene (under control of the HIV-1 LTR) was obtained from the NIH AIDS Research and Reference Reagent Program, Division of AIDS, NIAID, NIH (developed by Dr. John C. Kappes, and Dr. Xiaoyun Wu [79], [80]). The 293T cell line was obtained from Dr George Shaw (University of Alabama, Birmingham, AL). Cells were cultured at 37°C, 5% CO_2_ in DMEM containing 10% heat-inactivated fetal bovine serum (Gibco BRL Life Technologies) with 50 ug/ml gentamicin (Sigma) and disrupted at confluency by treatment with 0.25% trypsin in 1 mM EDTA (Sigma).

### Pseudovirus production

Selected envelope sequences were re-amplified from first round nested PCR products (described above) with PfuUltra II (Stratagene), purified by a Gel Extraction Kit (QIAGEN) and cloned into pcDNA3.1 (Invitrogen). The envelope plasmids were co-transfected into 293T cells using FuGENE 6 (Roche) with the pSG3ΔEnv backbone (obtained from the NIH AIDS Research and Reference Reagent Program, Division of AIDS, NIAID, NIH). Cultures were incubated for 48 hours to produce Env-pseudotyped viral stocks that were filtered through 0.45 µm and frozen in DMEM supplemented with 20% FBS. Mutant envelopes were generated with the QuikChange Lightning Kit (Stratagene) and confirmed by DNA sequencing (as above).

### Neutralization assay

The TZM-bl neutralization assay has been described previously [4], [81]. It measures a reduction in relative light units generated by a single round of infection in TZM-bl cells with Env-pseudotyped viruses after pre-incubation with monoclonal antibodies or a plasma sample of interest. Samples were serially diluted 1∶3 and the ID_50_ calculated as the dilution at which the infection was reduced by 50%.

### Protein expression and adsorptions

Plasmids encoding Histidine tagged recombinant envelope proteins were transfected into 293T cells using polyethylenimine 25 kDa (Polysciences). Recombinant proteins were expressed and purified as previously described [9]. Aliquots of 400 µg each were coupled to MyOne Tosyl-activated magnetic Dynabeads (Invitrogen) at 37°C pH 9.5 overnight, and then blocked with 0.5% BSA in 0.05% Tween20 PBS overnight at 37°C. Protein coupled beads were incubated with 200 µL of plasma (diluted 1∶20) for two hours at 37°C, then the beads were removed magnetically and the remaining plasma assessed for binding and neutralizing antibodies using ELISA and neutralization assays respectively.

### Enzyme Linked Immunosorbent Assay (ELISA)

Protein antigens were coated at 4 µg/mL onto high binding 96-well ELISA plates (Corning) overnight at 4°C. All subsequent steps were carried out in 5% fat-free milk, 0.05% Tween20 in PBS for 1 hour at 37°C. The plates were blocked and then probed with serial dilutions of the adsorbed plasma or specific monoclonal antibodies, biotinylated goat anti-human polyclonal antibodies (KPL), and an anti-biotin monoclonal conjugated to HRP (Calbiochem). Antigen-antibody complexes were detected by incubating with 100 µL 1-Step Ultra TMB-ELISA (Thermoscientific) for five minutes and then the reaction was stopped with 25 µL 1 M H_2_SO_4_. Absorbance was read at 450 nm on a VERSAmax tunable microplate reader (Molecular devices).

### Protein deglycosylation

Plasmid encoding the RHPA gp120 core was transfected with polyethylenimine-MAX 40 kDa (Polysciences) into GnTI(−/−) 293S cells and purified using two step lectin chromatography and Ni-NTA affinity chromatography. Protein was assessed for purity and conformation by SDS-PAGE and ELISA. 1 mg of the RHPA core gp120 was deglycosylated overnight at 37°C in 500 mM sodium chloride, 100 mM sodium acetate pH 5.5 with 0.5 U of Endo-H. Glycans were removed through buffer exchange into PBS using Vivaspin 20 mL concentrators (Sartorius stedim). Deglycosylation was confirmed by SDS-PAGE and sandwich ELISA (described above) using lectins as a capture protein.
